# Infusing Expert Knowledge Into a Deep Neural Network Using Attention Mechanism for Personalized Learning Environments

**DOI:** 10.3389/frai.2022.921476

**Published:** 2022-06-03

**Authors:** Ange Tato, Roger Nkambou

**Affiliations:** Artificial Intelligence Research Center, Department of Computer Science, Université du Québec à Montréal, Montreal, QC, Canada

**Keywords:** user modeling, deep learning, attention, socio-moral reasoning skill, logical reasoning skill, expert knowledge, hybrid neural networks

## Abstract

Machine learning models are biased toward data seen during the training steps. The models will tend to give good results in classes where there are many examples and poor results in those with few examples. This problem generally occurs when the classes to predict are imbalanced and this is frequent in educational data where for example, there are skills that are very difficult or very easy to master. There will be less data on students that correctly answered questions related to difficult skills and who incorrectly answered those related to skills easy to master. In this paper, we tackled this problem by proposing a hybrid architecture combining Deep Neural Network architectures— especially Long Short-Term Memory (LSTM) and Convolutional Neural Networks (CNN)—with expert knowledge for user modeling. The proposed solution uses attention mechanism to infuse expert knowledge into the Deep Neural Network. It has been tested in two contexts: knowledge tracing in an intelligent tutoring system (ITS) called Logic-Muse and prediction of socio-moral reasoning in a serious game called MorALERT. The proposed solution is compared to state-of-the-art machine learning solutions and experiments show that the resulting model can accurately predict the current student's knowledge state (in Logic-Muse) and thus enable an accurate personalization of the learning process. Other experiments show that the model can also be used to predict the level of socio-moral reasoning skills (in MorALERT). Our findings suggest the need for hybrid neural networks that integrate prior expert knowledge (especially when it is necessary to compensate for the strong dependency—of deep learning methods—on data size or the possible unbalanced datasets). Many domains can benefit from such an approach to building models that allow generalization even when there are small training data.

## 1. Introduction

Personalization (or adaptation) is a fundamental function when building adaptive learning systems. One of its advantages is to address a diverse audience (Birk et al., 2015). Adaptation has been widely considered in the field of AIED (*Artificial Intelligence In Education*) where one of the goals is to make intelligent tutoring systems (ITS) more effective. The adaptation of content and interactions based on a dynamic model of the learner remains one of the main issues addressed in this field (Woolf, 2010). Learning performance can be enhanced immensely by identifying the characteristics of students and adapting contents and presentation to better suit their needs (Tseng et al., 2008). Many techniques have been proposed to identify learners' characteristics during interactions with systems. Those techniques include Bayesian Knowledge Tracing (BKT), Deep Knowledge Tracing (DKT), or simply the use of neural networks for user modeling. The user (learner/student) model is an abstract description of the user in an environment (Bakkes et al., 2012). User modeling plays an important role in adaptive systems such as ITSs, serious games, MOOCs (Massive Open Online Courses), and even the applications and tools that surround us in our daily lives, such as social networks and cell phones. It is important to develop a solution that is able to model the user accurately from certain observations and thus be able to predict her/his needs, her/his behavior, etc. User modeling can include Knowledge Tracing which is considered the most popular approach for modeling learners. It aims at modeling how students' knowledge evolves during learning (Corbett and Anderson, 1994). The student's knowledge is modeled as a latent variable and is updated based on her/his performances on given tasks. It can be formalized as follows: given interactions *x*_1_, …, *x*_*t*_ of a learner on a particular learning task, how will she/he behave at *t* + 1? The goal being to estimate the probability *p*(*x*_*t*+1_|*x*_1_, …, *x*_*t*_).

Deep learning is an approach that has been successfully applied in many domains including images recognition (He et al., 2016), Natural Language Processing (Collobert and Weston, 2008) and more recently in education for knowledge tracing. Among the different deep learning architectures that exist, the one that fits well for knowledge tracing is the LSTM (Long Short Term Memory) as it can capture the sequential aspect of data useful for prediction. Hence, DKT (Piech et al., 2015) uses a LSTM to predict student performance based on the pattern of their responses sequentially over time. DKT traces knowledge at both the skill and the problem (item) levels and observes the correctness of each answer. At any time step, the input layer of the DKT is the student's performance on a single problem related to the skill that she or he is currently working on. In other words, the skill associated with and the correctness of the learner's performance on each item are used to predict how well she/he will perform on the next item (Zhang et al., 2017b). Rather than constructing a separate model for each skill as BKT does, DKT models all skills jointly (Piech et al., 2015; Khajah et al., 2016). It has been shown that DKT can robustly predict whether or not a student will solve a particular problem correctly given the accuracy of historic solutions (Wang et al., 2017; Zhang et al., 2017b).

However, they are biased toward the data they have seen before (it is a data-driven approach). Therefore, the generalization capability of a deep learning model depends on the training data. For problems (or skills) that are difficult to master which means the rare occurrence of correct answers, the model will be unable to accurately predict the student's knowledge. In the same vein, for skills that are easy to master which means the rare occurrence of incorrect answers, the model will also fail to accurately predict the student's knowledge. This problem is known in deep learning as the class imbalance problem (Huang et al., 2016) where there are fewer occurrences of data for a certain class (e.g., correct answers on skill difficult to master), which results in sub-optimal performance. A wrong user modeling can significantly affect the personalization process. In this paper, we propose to leverage this problem by enhancing deep learning models with expert knowledge added using the attention mechanism (Xu et al., 2015).

The main contribution of this work is to show how an original hybrid deep neural architecture infused with a priori expert knowledge through attention mechanism improves its generalization ability. The resulting architecture has proven its effectiveness for learner modeling and personalization in two educational software.

## 2. Related Work

### 2.1. Machine Learning and Deep Learning/AI Approaches for User Modeling

The use of machine learning and deep learning algorithms for user modeling purposes has attracted much attention in the past. In general, the growth in the volume of available data and the improvement of machine learning approaches are behind the recent boom of research in this domain. In data-driven approaches, researchers use machine learning and deep learning techniques to develop models to predict users' intentions and behaviors and extract user classes or behavioral groups from the interaction data collected in the system. Each user is thus assigned one or more classes of reactions and behaviors based on her/his actions. The most used algorithms in this context are generally K-means (Zakrzewska, 2008; Drachen et al., 2016; Troussas et al., 2020), deep neural networks (Demuth et al., 2014; Piech et al., 2015; Zhang et al., 2017a; Domladovac, 2021; Moon et al., 2021), naive Bayesian networks (Stern et al., 1999; Pardos and Heffernan, 2010), and SVMs (Support Vector Machine) (Hearst et al., 1998; Muyuan et al., 2004).

Missura and Gärtner (2009) developed a model to dynamically adapt the difficulty of a game based on the *clusters* extracted from the game data. They used the K-means algorithm to create *clusters* of users, each cluster representing a certain type of player. The adaptation model implemented here was implicit and controlling: each cluster corresponds to a certain level of difficulty. They used the SVM algorithm and a regression model in a prediction model to predict users' behavior, including actions taken, decisions made, and the cluster they might belong to. Once this cluster was determined, the difficulty was modified accordingly during the game. Ha et al. (2011) proposed a model of the player representing the goals he is trying to reach. To do so, they developed a framework based on the use of the Logical Markov Random Field (an undirected probabilistic graphical model with structures determined by first-order logic formulas) for goal recognition. Goal recognition is the task of inferring users' intended goals from observed sequences of actions. It was used in *Crystal island* (Rowe et al., 2009), a game-based learning environment where learners' goals are inferred through their responses to questions that the system asked narratively. Yannakakis and Hallam (2009) proposed a real-time adaptation model whose objective was to optimize user satisfaction. An artificial neural network and a parameter selection algorithm were applied to data from a survey (responses to questionnaires administered before and during the use of the system) and from the activity log (score, average response time, etc.), to generate a prediction model of user satisfaction. Drachen et al. (2012) proposed a grouping of different observable behaviors in the system. The goal was to obtain classes of behaviors to model the users. They use data mining algorithms including K-Averages and Simplex Volume Maximization (SIVM). Similarly, Gow et al. (2012) proposed a user model built from a semi-automatic and unsupervised learning approach combined with multi-class Linear Discriminant Analysis (LDA) (McLachlan, 2004) applied to the *logs*. Dass et al. (2021) have used the random Forest model to predict the dropout of students from a MOOC course given a set of features engineered from student daily learning progress.

The emergence of *big data* and machine learning techniques have opened up several possibilities for more accurate modeling of users especially in Educational Data Mining (EDM) domain. Bayesian and deep learning techniques are well-suited to the situation. They have allowed notable results in the field of behavioral modeling, particularly in knowledge tracing. Some authors have used Bayesian networks for modeling the knowledge acquisition process (Tato et al., 2017a; Kantharaju et al., 2018; Zhang and Yao, 2018). Several other authors have recently focused on knowledge tracing using deep learning architectures including recurrent neural networks (RNNs) and long and short-term memories (LSTMs) (Piech et al., 2015; Wang et al., 2017; Zhang et al., 2017b; Montero et al., 2018; Song et al., 2021; Xing et al., 2021) and other have focused on user modeling using the same techniques with some improvements (Yu et al., 2019; Nur, 2021; Yuan et al., 2021). It is worth noting that the use of Bayesian networks can be seen as a hybrid approach since the networks can be designed manually by domain experts (Conati et al., 1997; Horvitz et al., 1998; Rowe and Lester, 2010) or designed (architecture and probability tables) automatically from the data using machine learning approaches (Friedman et al., 1999; Tsamardinos et al., 2006).

Although data-driven methods hold great promise for automatically extracting relevant features, they can also extract meaningless information (Demuth et al., 2014). For example, these methods can extract irrelevant relationships such as a relationship between age and character choice in a game, since both pieces of information will tend to be repeated if multiple players of the same age often choose the same character. Classification, prediction, and clustering of behaviors in today's adaptive systems are becoming increasingly challenging due to the volume, high dimensionality, and multi-modality of data (Drachen et al., 2016). Also, the fundamental techniques used for learning in neural networks are not necessarily adapted for user modeling in EDM and do not take into account a priori and a posteriori knowledge. Therefore, it is necessary to rely on hybrid techniques able to take the best of both data-driven and theory-driven approaches.

### 2.2. Hybrid Neural Networks

Approaches using neural networks are called data-driven techniques since the training and the extracted model depend only on the training data. Their performance is highly dependent on the quantity and quality of the data. In general, the use of fully data-driven approaches requires the acquisition of a huge amount of data, which is not always practical or realistic for economic reasons or because of the complexity of the process involved. There are several domains where there is few training data but where a priori and/or a posteriori knowledge are available. This is particularly the case in the fields of education and the humanities. How can a purely data-driven but efficient architecture benefit from this knowledge often acquired over years of research? Such solutions combining data-driven and theory-driven approaches are considered hybrid. Other works have focused on hybrid neural networks but in terms of the combination of multiple neural architectures (Chen and Zhang, 2021; Sharma et al., 2021; Yan et al., 2021).

Towell and Shavlik (1994) defined hybrid learning techniques as “methods that use theoretical knowledge of a domain and a set of examples to develop a method for accurately classifying unseen examples during training.” A hybrid learning approach should learn more efficiently compared to approaches that use only one of the two main sources of information (data and theory). There is very little research on the combination of a priori/a posteriori knowledge and deep learning architectures. Towell et al. (which to our knowledge is one of the first research work to be interested in this question) have proposed a hybrid system called KBANN (Knowledge-Based Artificial Neural Networks). They map expert knowledge, represented in propositional logic, into neural networks and then refine this reformulated knowledge using back-propagation. Coro et al. (2013) combined neural networks with simulated expert knowledge. The simulated expert was used to generate some examples, which were then added to the training set of their neural network model. Zappone et al. (2018) recently did the same by combining expert knowledge and artificial neural networks (ANNs) to optimize wireless communication networks. They first obtained a training set from the expert knowledge and then trained the ANN on this generated training set. Finally, the pre-trained architecture was refined through a new training phase based on real measured data.

In educational domain, a priori and a posteriori knowledge are usually available and are used to build adaptive learning systems such as ITS (Intelligent Tutoring Systems). In other domains, knowledge may be available through books or already-built models, such as rule-based models. We believe that this knowledge, sometimes acquired during decades of intense research, cannot simply be ignored. Thus, we propose an approach that uses the attention mechanism (Xu et al., 2015) and capitalizes on the availability of these a priori and a posteriori data to reduce the amount of empirical data needed as well as the complexity of deep learning architectures. To the best of our knowledge, no research proposes to combine a priori and a posteriori knowledge with neural architectures using the attention mechanism. Moreover, no research in the educational domain, especially in user modeling, has addressed this issue despite the availability of knowledge. We show that the combination of knowledge-based and data-based methods using the attention mechanism is an appropriate solution for the design of hybrid deep neural networks with better generalization capability.

#### 2.2.1. A Priori and a Posteriori Knowledge

Sometimes a priori and a posteriori knowledge may be available, but it is not always sufficiently precise or is in a form that is not always easy to exploit. Nevertheless, even imprecise models can provide useful information that should not be discarded (Zappone et al., 2018).

In philosophy, a priori knowledge is defined as knowledge not gained through experience in contrast to a posteriori knowledge (Greenberg, 2010). In a priori and a posteriori knowledge, we include knowledge from experts. Expert knowledge represents the skills demonstrated by experts in a particular domain. In the educational domain, both a priori and a posteriori knowledge are generally available. For example, in Tato et al. (2017a), expert knowledge was used *via* a Bayesian network, designed entirely by domain experts to predict learners' logical reasoning skills. This knowledge is available in most cases in the form of written material such as books, experimental results, etc.

Our goal is to combine a priori and a posteriori knowledge with data-only techniques. We assume that the quality of the expert knowledge must be good in order for the proposed solution to be effective. We will have to make this knowledge interpretable and usable by a machine. A well-known approach is to build a rule-based system (Fisher et al., 1990; Cordón et al., 2001). For example, Van Melle (1978) used knowledge from experts to develop the MYCIN expert system. However, it can be difficult and tedious to operationalize expertise into rules. This is a task that typically requires the intervention of cognitive engineers. Also, these expertises are mostly buried in a written representation. What we propose is to use pre-designed models (e.g., rule-based systems, Bayesian networks, etc.) as is when available, or to use standard natural language processing and information retrieval techniques for knowledge extraction from text.

#### 2.2.2. Attention Mechanism

**Figure 1:**
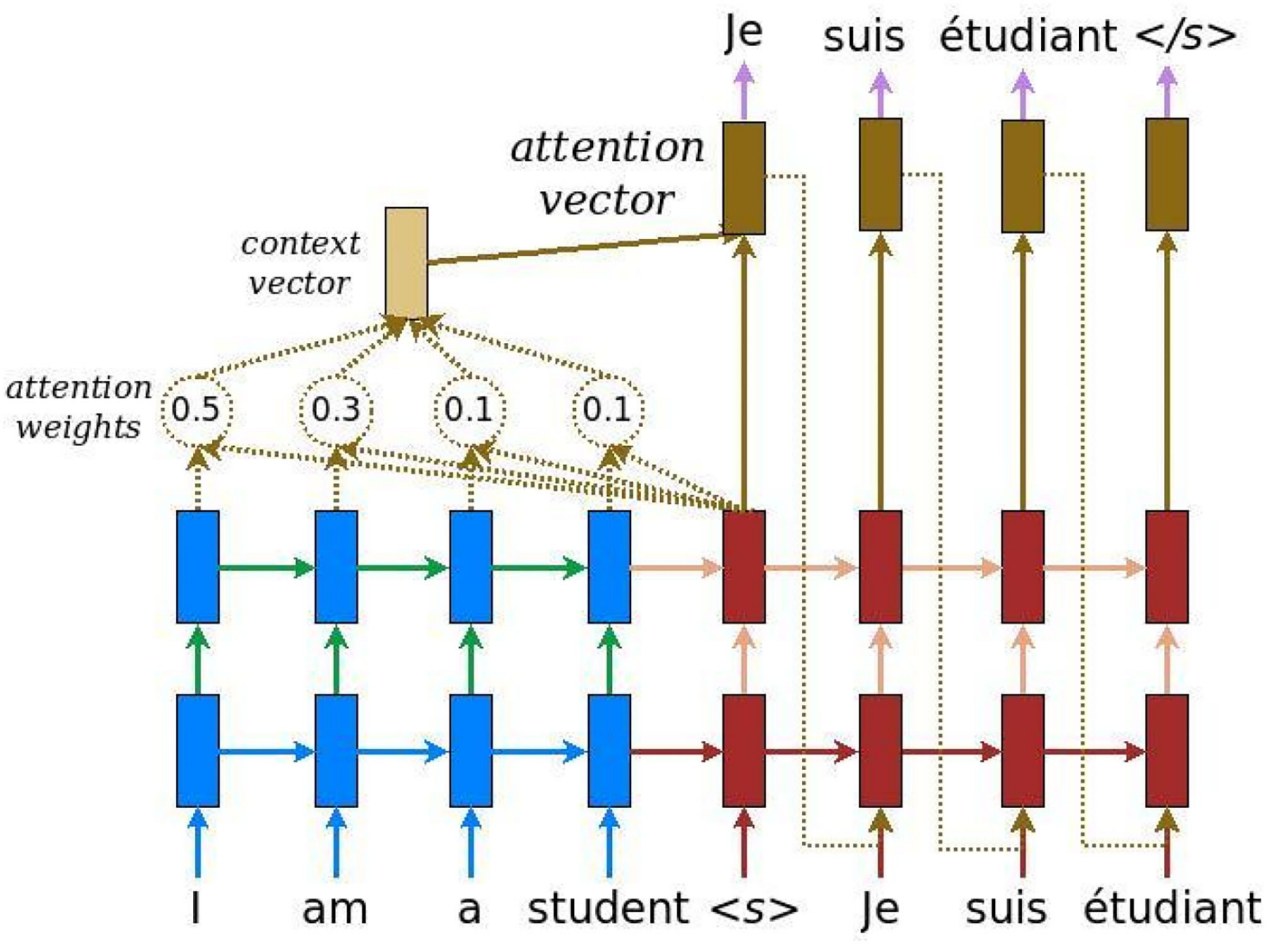
Attention mechanism. Example of translation from English (*I am a student*) to French (*je suis un étudiant*).

The attention mechanism allows a neural network to focus in part on a subset of information. Attention-based recurrent networks have been successfully applied to a wide variety of tasks: machine translation (Bahdanau et al., 2014; Liu and Chen, 2022), handwriting synthesis (Graves, 2013; Bhunia et al., 2021), speech recognition (Chorowski et al., 2015). Attention is a weight vector that allows neural networks to “intelligently” select the information contained in the input that is deemed important for decision making. The attention mechanism looks like a basic auto-encoder (see Figure 1^1^).

The principle is to connect a context vector between the encoder (in blue) and the decoder (in red). The context vector takes as input the outputs of all input cells to compute the probability distribution of the source elements for each output that the decoder wants to generate. This mechanism is similar to human attention, whose goal is to focus on a portion of current information that is deemed important for future decision-making. The context vector is constructed as follows: for a fixed target element (which represents a word in the Figure 1), a first loop is made on all the outputs of the hidden layers (states) of the encoders to compare the target and source states to generate scores for each state in the encoders. Then, the function *softmax* is used to normalize all the scores, which generates the probability distribution conditioned on the target states. Finally, weights are introduced to facilitate the learning of the context vector. Mathematically, here is how the attention vector is computed:


(1) $$\begin{array}{l} \alpha_{t,s}=\frac{\exp(score(h_{t},{\bar{h}}_{s}))}{\sum_{s^{\prime }=1}^{S}\exp(score(h_{t},\bar{h_{s^{\prime }}}))}\\ c_{t}=\sum_{s}\alpha_{t,s}\cdot {\bar{h}}_{s}\\ a_{t}=tanh(W_{c}[c_{t};h_{t}]) \end{array}$$

The variable *h*_*t*_ represents the output of the decoder at time t, *c*_*t*_ is the context vector and *a*_*t*_ is the attention vector. Here, the *score* is denoted as a content-based function. It evaluates how well each encoded input (*overlineh*_*s*_) matches the current output *h*_*t*_ of the decoder. The scores are normalized using the function *softmax* (α_*t,s*_). This score can be computed in different ways, among others by using the product: $score\left(h_{t},{\bar{h}}_{s}\right)=h_{t}^{⊺}\cdot {\bar{h}}_{s}$ (Luong et al., 2015). Finally, the variable *c*_*t*_ is the context vector.

## 3. Methodology

**Figure 2:**
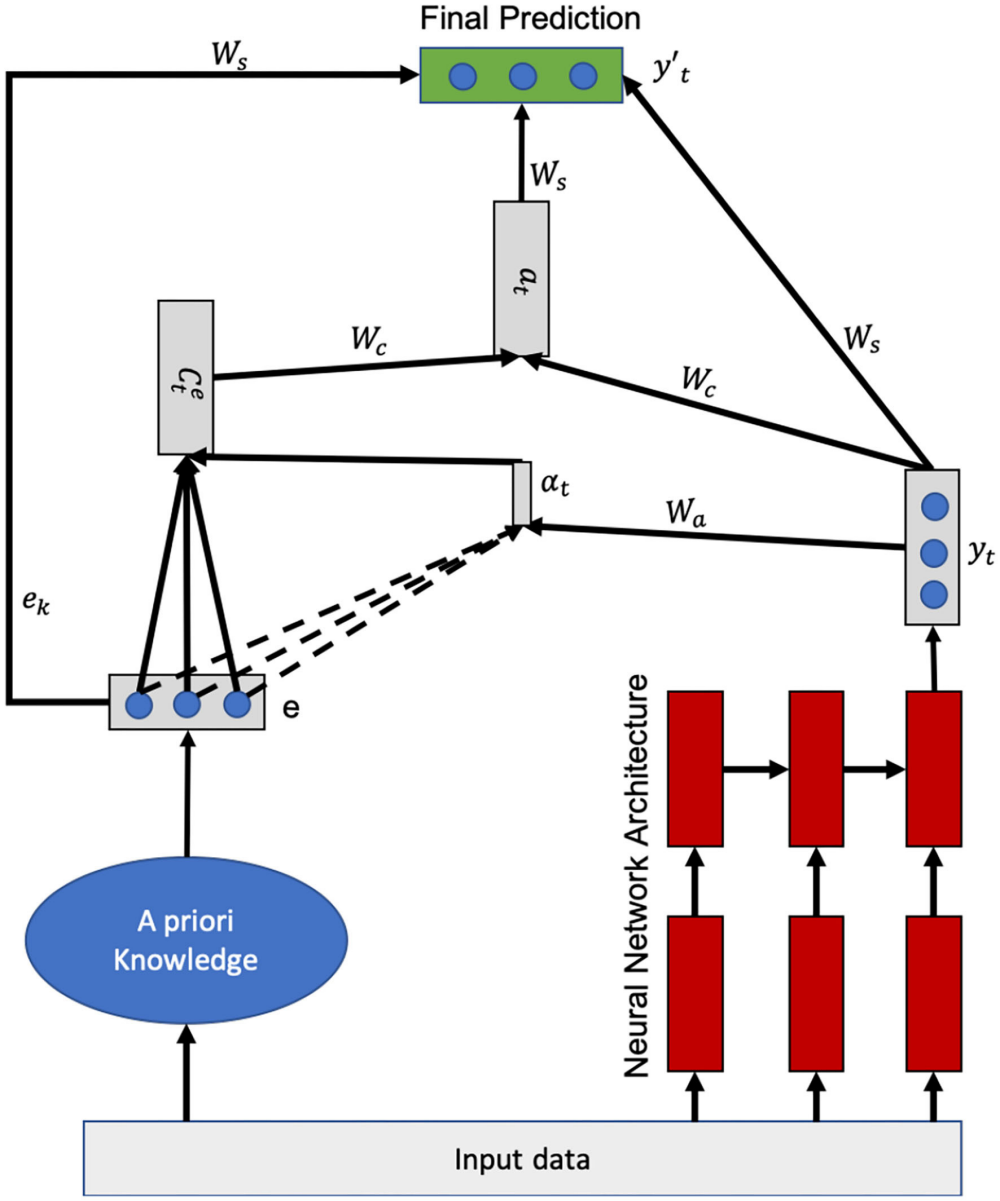
Global attentional hybrid model.

The internal architecture of neural networks makes it difficult to incorporate domain knowledge into the learning process (Lu et al., 1996). Our solution consists in forcing the model to pay attention to what the a priori and a posteriori knowledge *think* of the current input *x*. It thus aims to infuse the expert's final prediction into the system, rather than how this knowledge is processed by the expert. Since the attention mechanism (Xu et al., 2015) is a memory access mechanism as presented above, it is a perfect fit in this context where we want the model to have access to existing knowledge during learning. In other words, the neural network will “consult” this knowledge before making the final decision. The importance that the neural model will give to what this knowledge predicts as output is computed using attention weights (*W*_*a*_ and *W*_*c*_, see Figure 2). As the network learns, it will know how much importance it will give to each of the predictions coming from the knowledge based on the trials/errors it will have committed. *W*_*a*_ corresponds to the weights calculating the importance of each feature learned by the neural architecture (*y*_*t*_) compared to each feature extracted from the knowledge. *W*_*c*_ represents the weights measuring the importance of the predictions made from the knowledge (*via* the context vector) and the learned features (*y*_*t*_) for the estimation of the final prediction vector (see Figure 2). Thus, the model will focus on what the expert says, including a priori and a posteriori knowledge, before making a decision. By merging the expert knowledge with the neural architecture using the attention mechanism, the model iteratively processes the knowledge by selecting the relevant content at each step. In the attentional mechanism presented by Luong et al. (2015), specifically the global attentional model, the attentional vector is calculated from the target hidden state *h*_*t*_ and the input hidden state. Instead of the input hidden state ${\bar{h}}_{s}$ as presented above, we will have the data from the expert knowledge that will be used to compute the context vector *C*_*t*_ which we will call the expert-side context vector (see Figure 2 in Luong et al., 2015). Thus, given the hidden state *y*_*t*_ which is the final prediction of the neural model, and the expert-side context vector $c_{t}^{e}$, we use a concatenation layer to combine the information from the two vectors to produce the attentional hidden state *a*_*t*_ as follows:


(2) $$\begin{array}{l} a_{t}=tanh\left(W_{c}\left[c_{t}^{e};y_{t}\right]\right) \end{array}$$

The attentional vector *a*_*t*_ together with the prediction made from the knowledge *e* and the hidden state *y*_*t*_ (the prediction) of the neural model are then sent to a dense layer to produce the expected result $y_{t}^{\prime }$. The expert-side context vector is computed as follows:


(3) $$\begin{array}{l} score(e_{k},y_{t})=e_{k}\cdot y_{t}\cdot W_{a}+b\\ \alpha_{t,k}=\frac{\text{exp}(score(e_{k},y_{t}))}{\sum_{j=1}^{s}\text{exp}(score(e_{j},y_{t}))}\\ c_{t}^{e}=\sum_{k}\alpha_{t,k}\cdot e \end{array}$$

Where 1 ≤ *k* ≤ *s*, *e* is the prediction made from the a priori and a posteriori knowledge, *y*_*t*_ is the current prediction made by the neural architecture and *s* is the size of the predicted vector (the number of classes to predict). The variable *e* represents a vector of length equal to the size of the vector to be predicted where each entry represents the probability that the entry belongs to each of the classes according to the expert's knowledge. The variable *e*_*k*_ represents of size 1 and *W*_*a*_,*W*_*c*_,*W*_*s*_,*W*_*s*_,*y*_*t*_,*e*, *a*_*t*_ are of size *s*. The score as mentioned above is a content-based vector that computes the correlation (alignment score) between the expert knowledge and the latent *features* learned by the neural architecture. This parameter defines how expert knowledge and latent features learned from the data are aligned. The model assigns a score α_*t,k*_ to the pair of features at position *t* and the expert knowledge (*e*_*k*_, *y*_*t*_), based on their correspondence. The set of α_*t,k*_ are weights defining how much each feature of the data from the expert should be considered for each output (final prediction). Figure 2 shows in detail this global process.

Figure 2 presents the attention mechanism applied to a priori knowledge. The operating principle is simple and intuitive:

- In the first step, the input data *x* is processed by the expert, i.e., the membership of *x* to one of the classes to be predicted is evaluated from the a priori and a posteriori knowledge. The same data *x* is also processed by the neural architecture in parallel. In the Figure 2, we have taken the example of an LSTM but it can be replaced by a CNN or a simple DNN.
- What we call “A priori knowledge” in the Figure 2 can be replaced by any process simulating the expert's functioning or any process simulating a priori and a posteriori knowledge. It can be a rule-based engine or a Bayesian network for example. The output of the a priori knowledge is a vector representing a probability distribution where each of the inputs is the probability that the data *x* belongs to each of the classes to predict. For example, if we want to predict that an image is either a cat or a dog, then the knowledge output is a vector of size two whose first value represents the probability that the image belongs to the cat class and the second is the probability that the image belongs to the dog class. In our proposed architecture, we have assumed that the size of the expert's output vector is equal to the output vector of the final prediction. However, it can be of different sizes.
- We have considered only the output of the last cell (*y*_*t*_) of the LSTM, but one could also concatenate all hidden states (*h*_0_, …, *h*_*t*_) for the calculation of the context vector.
- Once the expert vector (*e*) and *y*_*t*_ are estimated, the architecture determines the expert context vector (called *c*^*e*^). This vector represents the relevant information (features) extracted from the combination of the expert knowledge and the information extracted by the neural architecture. It is computed according to the formula presented above.
- Once the expert context vector is estimated, the attention vector (*a*) is computed from the concatenation of the expert context vector and *y*_*t*_ (see computation formula above).
- For the estimation of the final prediction vector $y_{t}^{\prime }$, there are several possible solutions. The first one (which is presented in the Figure 2) would be to concatenate the expert vector (*e*), the data learned by the neural architecture (*y*_*t*_), and the attention vector *a*_*t*_ and then pass the vector obtained into an activation function to compute the final vector. Other solutions would consist in, not including one or both vectors *e* and *y*_*t*_ in the concatenation.

We have presented our approach using an LSTM as an example, but in the case of a CNN, the *y*_*t*_ would represent the final output of the network that has been flattened. The proposed hybrid model will allow taking into account the a priori and a posteriori knowledge of the experts for the modeling of the users. One of the uses of such a model is knowledge tracing or skill prediction, which we will present in the next sections dedicated to the application of our solution in real cases.

## 4. Experiments

Our goal is to create an accurate user model or a good model for the prediction of skills, in order to improve personalization in adaptive learning systems.

### 4.1. Logic-Muse

Logic-Muse is a web-based Intelligent Tutoring System (ITS) that helps learners improve logical reasoning skills. Logic-Muse includes a learning environment which uses various meta-structures to provide reasoning activities on various contents (Nkambou et al., 2015). The expert model implements logical reasoning skills and knowledge as well as related reasoning mechanisms (syntactic and semantic rules of the given logical system). The model of both valid and invalid reasoning rules is encoded as a set of production rules. In addition, the semantic memory of the target logic (the logic behind the reasoning) is implemented through a formal OWL ontology and connected to the inference rules. The first version of Logic-Muse focuses on propositional logic. Logic-Muse learner model goal is to represent, update and predict the learner state of knowledge based on her/his interaction with the system. It has multiple aspects including the cognitive part that essentially represents the state of the learner's knowledge (mastery of the reasoning skills in each of the six reasoning situations that have been identified thanks to the experts). The cognitive state is generated from the learner behavior during his interactions with the system, that is, it is inferred by the system from the information available. It is supported by a bayesian network (Tato et al., 2017a) based on domain knowledge, where influence relationships between nodes (reasoning skills) as well as prior probabilities are provided by the experts. Some nodes are directly connected to the reasoning activities (items). The skills involved in the BN are those put forward by the mental models theory to reason in conformity to the logical rules. These skills include the inhibition of exceptions to the premises, the generation of counterexamples to the conclusion and the ability to manage all the relevant models for familiar, contrary to fact (counterfactual) and abstract situations (Markovits, 2013). There are 16 skills directly observable that are linked to the exercises (see Table 1) and 12 latent skills. 48 exercises on logical reasoning were used in this study.

**Table 1:** Distribution of responses over skills.

**Skills**	**Skill description**	***N***	**Average**	**Standard dev**
MppFd	Modus ponendo ponens with few disabling conditions	294	**0.9456**	0.16078
MppMd	Modus ponendo ponens with many disabling conditions	294	0.898	0.23726
MppCcf	Modus ponendo ponens at the counterfactual level	294	**0.907**	0.2394
MppA	Modus ponendo ponens at the abstract level	294	**0.9615**	0.16066
MttFd	Modus tollendo tollens with few disabling conditions	294	0.8435	0.26646
MttMd	Modus tollendo tollens with many disabling conditions	294	0.7925	0.29985
MttCcf	Modus tollendo tollens at the counterfactual level	294	0.7494	0.33326
MttA	Modus tollendo tollens at the abstract level	294	0.8401	0.28974
AcMa	Affirmation of the consequent with many alternatives	294	**0.424**	0.38072
AcFa	Affirmation of the consequent with few alternatives	294	**0.3039**	0.3652
AcCcf	Affirmation of the consequent at the counterfactual level	294	**0.3345**	0.40801
AcA	Affirmation of the consequent at the abstract level	294	**0.2823**	0.41038
DaMa	Denial of the antecedent with many alternatives	294	**0.407**	0.37389
DaFa	Denial of the antecedent with few alternatives	294	**0.3027**	0.35081
DaCcf	Denial of the antecedent at the counterfactual level	294	**0.381**	0.40662
DaA	Denial of the antecedent at the abstract level	294	**0.305**	0.42077

*Skills difficult to master (Average <0.4) and skills easy to master (Average > 0.9) are in bold*.

For this first system, the goal is to train and test the DKT model enhanced with our attentional expert knowledge for knowledge tracing. DKT is a LSTM which take sequences of exercise-performance (*e*_*t*_, *p*_*t*_) pairs presented one trial at a time. The model then predict the knowledge state, based on the current hidden state. The hidden layer of the LSTM represents the latent encoding of knowledge state, based on the current input and previous latent encoding of knowledge state. It represents the latent knowledge state of student resulted from his past learning trajectory.

#### 4.1.1. Bayesian Network as Attention in DKT

Bayesian Network (BN) is a graphical model used to model processes under uncertainty by representing the relationship between variables in terms of a probability distribution (Russell and Norvig, 2016). BN allows inferring the probability of mastering a skill from a specific response pattern (Nkambou et al., 2010). The structure and the parameter or probability distributions are provided by experts or learned using algorithms such as Expectation-Maximization. It has been successfully used to model knowledge state of learners (Martin and VanLehn, 1995; Nguyen and Do, 2009; Tato et al., 2016) or learner affect (Sabourin et al., 2011). There are many contexts where a lot of data or expert knowledge (e.g., medicine, Flores et al., 2011) is available to build BN. How the DKT can benefit from that? In contexts where expert knowledge is available, we wanted to take advantage of that. We also articulate that, as expert knowledge is not biased toward rare data, the model could benefit from that when making predictions.

In Logic-Muse BN, there are 16 nodes directly observable (representing skills linked to exercises) and 12 latent nodes representing knowledge that are a combination of observable skills. At each time *t*, the BN predicts the probability of mastering each of the 16 skills for the current student based on its responses to past exercises. The output of the prediction represents a vector of 16 entries which is the vector *e* (expert knowledge in Figure 2) in the attentional hybrid model. By integrating the expert knowledge (here a BN) in the DKT using the attention, the model iteratively processes the a priori knowledge by selecting relevant content at every step.

#### 4.1.2. Dataset

There is a total of 294 participants who participated in this first study. They all completed the 48 logical reasoning exercises. In our dataset, each line of data represents each participant (a total of 294 data and a sequence length of 48). The exercises were encoded using skills that are directly observable, which means that the questions related to the same skill are encoded with the same Id (1–16). The skills with few data are determined by a comparison of the average of correct answers obtained for each skill. In Table 1, we averaged all the answers on each skill. The skills difficult to master are those with the lowest average value and the skills easy to master are those with the highest average. Since the LSTM only accepts a fixed length of vectors as the input, we used one-hot encoding to convert student performance into a fixed length of vectors whose all elements are 0 except for a single 1. The single 1 in the vector indicates two things: which skill was answered and if the skill was answered correctly.

#### 4.1.3. Results

To assess our proposed solution, we ran 2 models: the DKT and the DKT with a priori knowledge (DKT+BN). We used 20% of the data for testing and 15% for validation. The BN alone gave 69% of global accuracy. The result is evaluated using the *F*1*score* on each skill (treated as 2 classes—correct and incorrect answers) being predicted and the overall accuracy. The models were evaluated in 20 different experiments (the same experiment was repeated 20 times, this is to ensure that there is no randomness in the presented results) and the final results were averaged. In all our experiments, we set λ_1_ and λ_2_ = 0.10. Our implementation of the DKT+BN model in Tensorflow using Keras backend was inspired by the implementation^2^ done by Khajah et al. (2016) Our code is also available on GitHub^3^ for further research.

**Figure 3:**
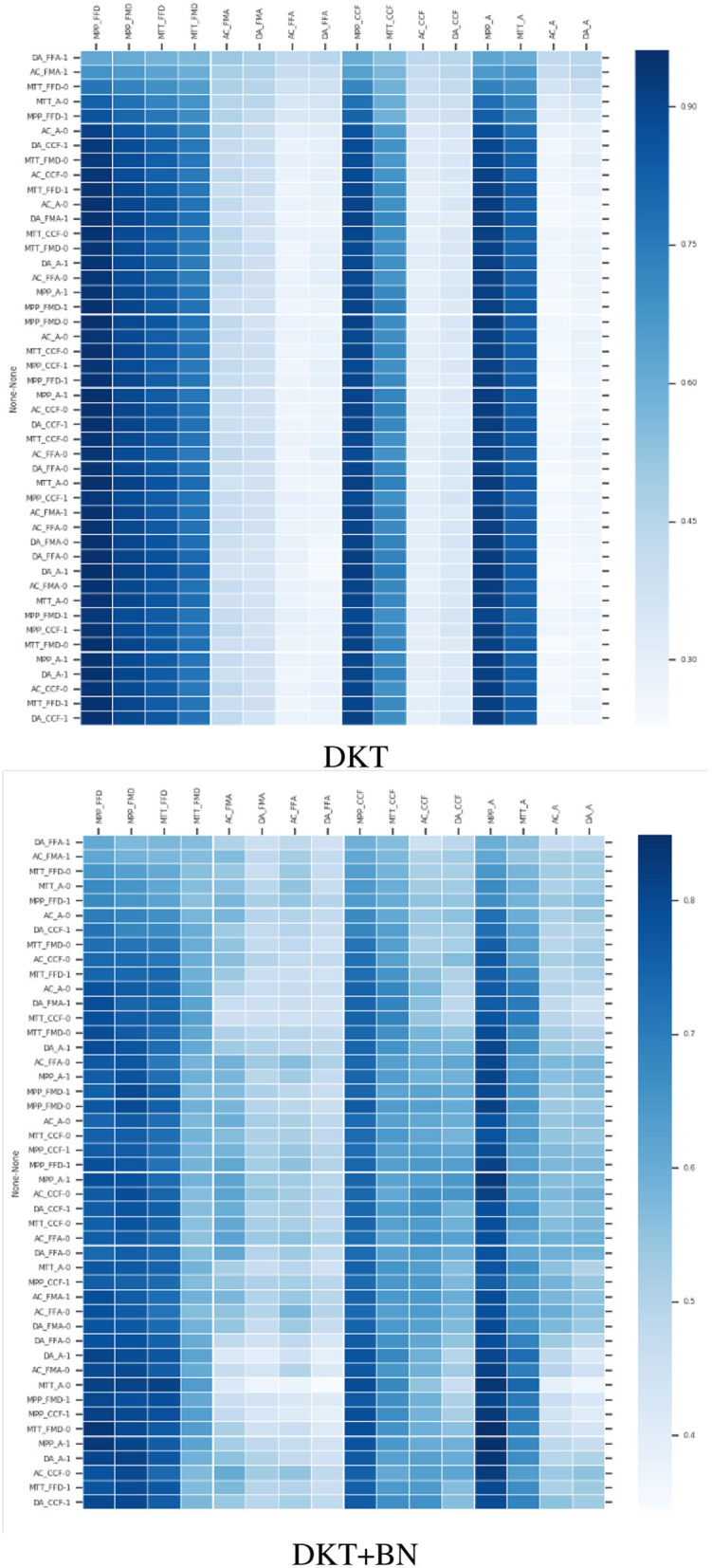
Heatmaps illustrating the prediction with the 2 models on the same student. DKT is unable to make accurate predictions on skills with little data, especially skills that are difficult to master. The label in the vertical dimension refers to the input fed into the models at each time step. The color of the heatmap indicates the predicted probability that the student will correctly answer a question related to a skill in the next time step. The darker the color, the higher the probability. Here, the student gave 2 out of 3 correct answers on AC_FMA and we see that the DKT+BN is able to track down that information compared to the DKT.

The results are shown in Tables 2, 3 and in Figure 3. As expected, the new DKT enhanced with BN outperforms the state-of-the-art model for predicting skills with few data. For skills that are easy to master (e.g., MPP type skills), all the models always predict that students will give correct answers (the F1score of incorrect answers is 0 for DKT and almost 0 for the other model). This is because, on the 294 data, we have for example only 6 incorrect answers for the MPP_FFD skill. We tested the models with high values for λ_2_ and we get values of f1score equal to around 0.6 for correct answers and around 0.7 for incorrect answers. This result may be satisfactory in other contexts but in the context of logical reasoning where it is established that the MPP type skills are always well-mastered, 0.6 as an f1score for correctly predicting a correct answer is not acceptable. That is why we kept λ_2_ =0.10. However, the solution stays valid for data where the ratio *r* = number of correct answers/ number of questions answered or *r* = number of incorrect answers/ number of questions answered on a skill is not too small (as in this case) and is less than 0.5. For skills that are difficult to master, there is a huge difference between the DKT and our proposed model. DKT is unable to track correct answers on skills difficult to master. This behavior cannot be accepted as the knowledge tracing of students that perform well on those skills will fail. Thus it is important to make sure that the final model is accurate for all the skills. On skills with few samples, we can notice that the DKT + BN still behaves better than DKT.

**Table 2:** The DKT and the DKT+BN on skills that are difficult to master.

**F1score**	**AcMa**		**DaMa**		**AcFa**		**DaFa**		
DKT	0.74	0.09	0.72	0.0	0.79	0.0	0.79	0.0	
DKT+BN	**0.70**	**0.64**	0.78	0.43	**0.86**	**0.74**	**0.80**	**0.47**	
**F1score**	**AcCcf**		**DaCcf**		**AcA**		**DaA**		**Accuracy**
DKT	0.79	0.0	0.73	0.0	0.80	0.0	0.79	0.0	**0.74**
DKT+BN	**0.87**	**0.80**	**0.82**	**0.72**	**0.83**	**0.58**	**0.88**	**0.80**	**0.82**

*We repeated the experiments 20 times. The last column is the overall accuracy of the model. For each skill, the first column corresponds to the value of the f1score for predicting that the skill was incorrectly answered and the second column to the value of the f1score for predicting that the skill was correctly answered. The best ratio for each skill is in bold*.

**Table 3:** The DKT and the DKT+BN on Skills that are easy to master.

**F1score**	**MppFd**		**MppMd**		**MttFd**		**MttMd**	
DKT	0.0	0.97	0.0	0.94	0.0	0.91	0.0	0.90
DKT+BN	0.0	0.95	0.0	0.97	0.0	0.89	0.19	0.88
**F1score**	**MppCcf**		**MttCcf**		**MppA**		**MttA**	
DKT	0.0	0.96	0.0	0.87	0.0	0.96	0.0	0.90
DKT+BN	0.0	0.97	0.12	0.85	0.0	0.99	0.1	0.89

*We repeated the experiments 20 times*.

During our experiments, we noticed that predictions are not sometimes consistent with reality as other works have also highlighted (Yeung and Yeung, 2018). The model fails to reconstruct the observed input. As a result, even when a student performs well on a skill, the prediction of that skill's mastery level decreases instead, and vice versa. Also, the predicted performance across time steps is not consistent. As seen in Figure 3—DKT, when a student gives correct answers to a skill *k*, the DKT does not sometimes update the current state of knowledge on that skill (the skill stays low or is updated very slowly). The problem can be addressed by adding regularization terms to the loss function of the original DKT as suggested by Yeung and Yeung (2018). It can also be partially solved when adding the apriori knowledge as we noticed during our experiments (Figure 3—DKT+BN). However, we stay confident in the fact that if the apriori knowledge is more accurate (which is not our case as the accuracy of the BN is 0.65) we will get better results.

### 4.2. MorALERT

#### 4.2.1. Context

Socio-Moral Reasoning (SMR) is a socio-cognitive construct essential for decision-making, as well as social interaction adaptation. It is commonly defined as “how individuals think about moral emotions and conventions that govern social interactions in their everyday lives” (Beauchamp et al., 2013). Being able to predict and diagnose one's socio-moral reasoning skill level (or ability) is a key step for quantifying peoples' social functioning and can be used to identify those at risk for maladaptive social behavior. This diagnosis could help orient people toward appropriate services or provide adequate support to improve this skill's development. The Socio-Moral Reasoning Aptitude Level (SoMoral) (Dooley et al., 2010) task is a computer-measured walkthrough in which children and adolescents are presented with visual social dilemmas from everyday life. They are then asked to verbalize how they would react in this situation, justifying their answer. The participants' answers are recorded verbatim in transcripts that are subsequently scored manually by experts using a moral-maturity coding scheme inspired by the Kohlberg's theory of moral development (Kohlberg, 1984). Verbatims are short or long text containing at least one sentence. Each socio-moral reasoning rating was well-documented by experts using annotated data. The goal was to put together these two kinds of data to build an accurate model for the automatic prediction of reasoning skill levels. Using annotated data only would not give us good results in this context. Therefore we propose a hybrid model that combines expert knowledge with DNN (Deep Neural Networks) architectures (especially CNN and LSTM) using the attentional mechanism (Xu et al., 2015) for predicting the level of SMR skill of an individual based on his justifications when solving socio-moral dilemmas. The developed solution extends the learner/player model in a serious game called LesDilemmes (Tato et al., 2017b).

#### 4.2.2. MorALERT: A Serious Game for Improving Socio-Moral Reasoning Skills

**Figure 4:**
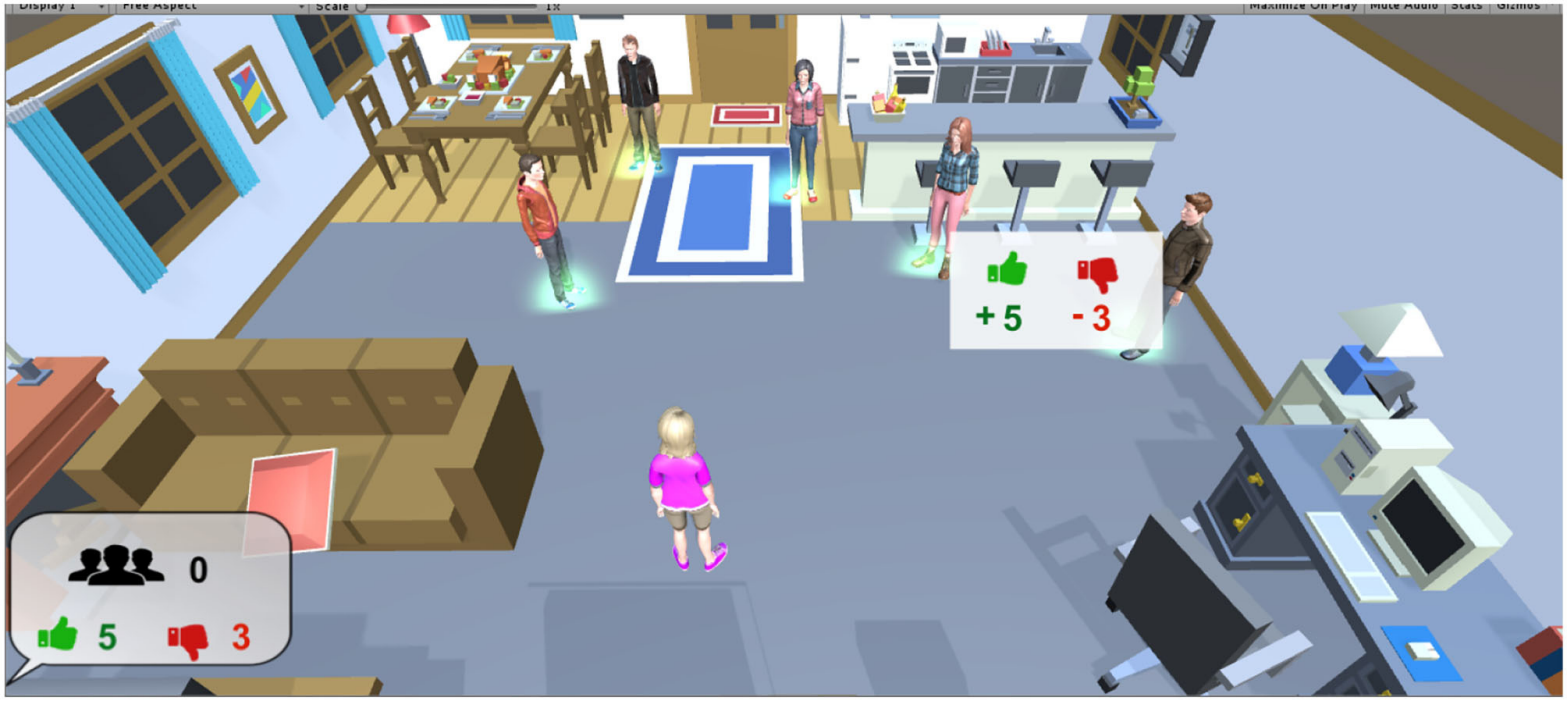
MorALERT serious game.

The proposed hybrid model has been tested for the automated scoring mechanism in a serious video game called MorALERT (previously called LesDilemmes) (Tato et al., 2017b). MorALERT is a first-person serious game aiming to assess and train the player on social reasoning skills. MorALERT is a virtual learning environment offering an emotionally, socially, and cognitively rich interactive context. Players face different socio-moral dilemmas within a 3D environment (see Figure 4) in which they have to make decisions and are asked to provide oral justifications for their choices. They can also ask the opinions of virtual friends (Non-Player Characters) in the game. At the beginning of the game, each Non-Player Character (NPC) is assigned an SMR maturity level. The NPCs' opinions are then selected from previously recorded transcripts corresponding to the different moral maturity levels according to the coding scheme (SoMoral, Beauchamp et al., 2013). The learner (player) model implemented in the learning environment includes three key dimensions: the affective state, the cognitive profile, and, the socio-moral reasoning profile. Therefore, socio-moral reasoning skill is part of the player model implemented in the game. As stated in Birk et al. (2015), a learner/player model that can accurately represent the learner longitudinally in a game leads to efficient adaptation, which in turn helps increase the player's satisfaction and motivation. To this end, it is important to ensure the effectiveness of the learner/player model before deploying the system for real uses. One goal in MorALERT was to build an effective model of the socio-moral facet of the learner/player. Defining an individual's socio-moral reasoning level necessitates an examination of the verbal justifications he provided when solving the dilemmas. This involves the implementation of a model that automatically measures and predicts this data during the game. We possessed a dataset of verbatim coming from the SoMoral experimentation already annotated by experts and a description (a paragraph with key concepts) associated with each different level (or class) of maturity. The experiment in this second context aims to implement our solution so that we can accurately assess the socio-moral reasoning skill level of a player based on his verbatim.

#### 4.2.3. Socio-Moral Reasoning Skill Levels

The original SoMoral task includes five different levels of socio-moral reasoning skill (Beauchamp et al., 2013): (1) Authoritarian-based consequences, (2) Egocentric exchanges, (3) Interpersonal Focus, (4) Societal Regulation, and (5) Societal Evaluation. Transition levels (i.e., 1.5, 2.5, 3.5, 4.5) are used to account for verbatim that provides elements of two different reasoning stages and show a sequential progression from one stage to another. Occasionally, a verbatim is assigned to two different closed levels (with a maximum deviation of 1) when two independent experts annotate the data for inter-reliability purposes.

A first convolutional neural network has been proposed, to automatically detect the player's socio-moral reasoning level. Despite the high accuracy reported (92%) (Tato et al., 2017b), the model was not able to predict when a person has a socio-moral reasoning level of 2, 3, or 5. All the predictions were either level 1 or 3 due to an unbalanced dataset. Our new model addresses this issue in order to ensure the accuracy of the learner model for better support of the learning process in the serious game.

#### 4.2.4. Hybrid Architecture for Socio-Moral Reasoning Skill Prediction

##### 4.2.4.1. Expert Knowledge

**Figure 5:**
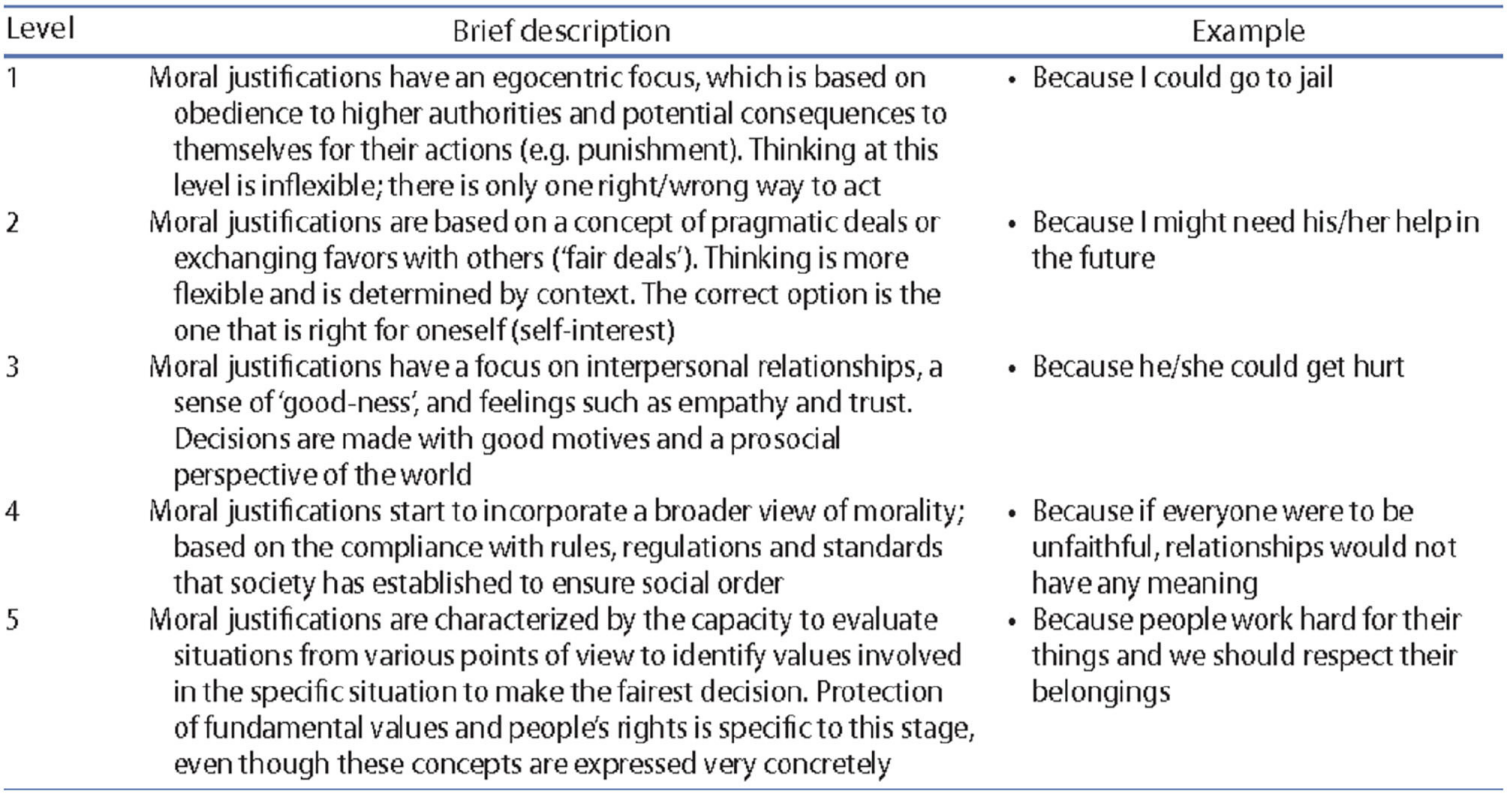
Brief description of SoMoral coding and examples (Chiasson et al., 2017).

In our context, where we only have access to description (in textual form) of each of the levels (see Figure 5, Chiasson et al., 2017), we employed two different techniques related to IR (Information Retrieval) and NLP (Natural Language Processing): the Word Movers' Distance to compare the meaning of texts, n-grams, and stemming to test whether verbatim contain those specific elements extracted for each level. We explain each of the techniques in further details in the following section.

##### 4.2.4.2. Word Mover's Distance

Word Mover's Distance (WMD) (Kusner et al., 2015) is a machine learning technique that allows submitting a query and returning the most relevant documents. It is used to assess the “distance” between two documents in a meaningful way, even when they have no words in common. The assumption is that similar words should be linked to similar vectors. Instead of using Euclidean Distance and other bag-of-words based distance measurement, they proposed to use word2vec vector embeddings of words to calculate similarities. It has been shown to outperform many of the state-of-the-art methods in k-nearest neighbors classification by normalizing Bag-of-Words and Word Embeddings to calculate the distance between documents.

We used the WMD method to build the first part of the expert knowledge content that will be combined with the DNN. Each description of each socio-moral reasoning skill level as well as each verbatim is considered as a document. We transformed each document to their word2vec representation using pre-trained word2vec models for French.^4^^,^^5^ The vectors have then been normalized so that they could all have equal length. We calculated the similarity between each verbatim and each description (5 descriptions) using gensim Wmd-Similarity tool.^6^ For each verbatim this calculation provided us with a vector of length 5 where each entry is the similarity between the verbatim and the description of the corresponding socio-moral reasoning skill level. The values range between 0 and 1.

##### 4.2.4.3. Bi-gram and Stemming

N-gram is a contiguous sequence of *n* items from a given sample of text or speech. We extracted n-grams (uni and bi grams) from the textual description of levels, which gave us a list of n-gram for each reasoning level. We also generated a list of synonyms of all keywords (extracted manually) included in the descriptions. For each verbatim, we counted the number of times each n-gram of each level appeared within the text.

Stemming is the process of reducing inflected (or sometimes derived) words to their word stem, base or root form. We applied this technique to all the words in the synonyms list generated and for each word in verbatim. Also, for each verbatim, we counted the number of times each synonym of each level appeared within the text. The sum of the vectors generated by this process and the previous one (n-grams) gave us, for each verbatim, a vector of size 5 where each entry represents the number of n-grams and synonyms of each level found in the verbatim. We finally applied the softmax function to the resulting vector which was added to the one generated by the WMD method.

#### 4.2.5. Experiments

In order to empirically evaluate the proposed solution in this second context, we investigated six different models: a CNN (cnn-only), an LSTM (lstm-only), a CNN where expert knowledge was concatenated to the features learned (cnn-expert), an LSTM where expert knowledge was concatenated to the features learned (lstm-expert) and finally the cnn-expert-att (a CNN where expert knowledge was concatenated to the features learned + knowledge-based attention) and lstm-expert-att (a LSTM where expert knowledge was concatenated to the features learned + knowledge-based attention). We used the same parameter initialization for all the models. Adam (Kingma and Ba, 2014) was used as the optimizer with a learning rate set to 0.001. The algorithms were implemented using Keras^7^ and the experiments were done using the built-in Keras models, making only small edits to the default settings.

##### 4.2.5.1. Data Pre-processing

The dataset consists of a benchmark of 731 verbatim (written in French) manually annotated by experts. Verbatims are not equally distributed between classes (unbalancing data). Table 4 shows the distribution of data where for example the classes 4 and 5 have a small number of verbatims than others (1,2 and 3). In fact, level 5 being the highest level of maturity, it is rare to come across people of this level. This means that some of those classes have very few examples to learn from. On the 731 verbatims, there were 53 of them that were classified as 0, which means that the verbatim does not represent one of the socio-moral reasoning skill levels. We do not consider these cases in our study, which reduces our corpus to 678 verbatims. We also limited our prediction problem to 5 levels of socio-moral reasoning skills: 1, 2, 3, 4, and 5. The verbatims belonging to intermediary levels 1.5, 2.5, 3.5, and 4.5 were added to verbatims belonging to levels 2, 3, 4, and 5, respectively.

**Table 4:** Distribution of verbatims between classes.

**Class**	**Frequency**	**Class**	**Frequency**
1	232	1.5	11
2	76	2.5	29
3	207	3.5	31
4	40	4.5	3
5	49		

##### 4.2.5.2. Deal With Unbalanced Data

**Figure 6:**
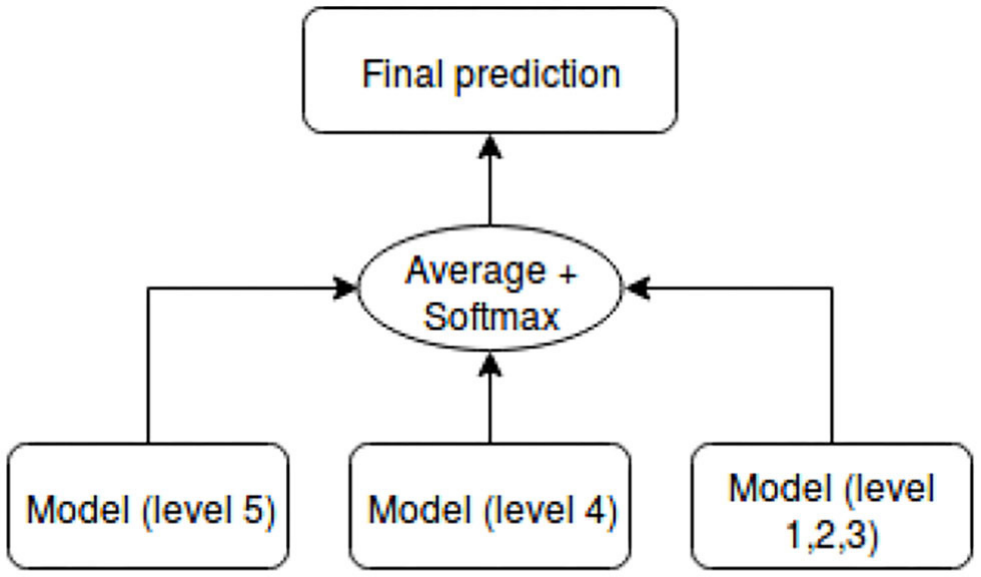
Final model for predicting socio-moral reasoning skill levels.

As shown in Table 4, the dataset is unbalanced. The first models we built were biased toward classes that had more samples. Therefore, we applied some techniques that aimed to balance the dataset. The first technique we had chosen is the penalization of models (cost-sensitive models, Shi et al., 2015). It works as follows: we penalize the classification by imposing an additional cost on the models for making classification mistakes on the minority class during training. The penalties can bias the model to pay more attention to the minority classes. However, this technique was unsuccessful because the results were no better than when using the original dataset. In fact, the generated solutions were able to classify instances from the minority classes correctly, but less capable of classifying instance from the majority classes. The second technique we used aimed at re-sampling the dataset (Ghosh et al., 2017). With this technique, the models were insured to not be biased toward one class. The dataset was re-sampled by using the oversampling technique, which consists in randomly duplicating samples from classes with few examples, in order to match the number of samples of other classes. This second technique did not work as well. Instead of playing with the data, we found a solution based on training multiple models that are specialized in the prediction of specific socio-moral levels. We used ensemble methods to combine results from those models and produce the final prediction. Ensemble methods (Dietterich, 2000) are techniques that create multiple models and then combine them to produce improved results. Voting and averaging are two of the easiest ensemble methods. They are both easy to understand and implement. Voting strategy was selected, as it is well-suited for classification problems. The architecture of the final model is presented in Figure 6. In Figure 6, each sub model is specialized for the prediction of levels specified between parenthesis. Each model is either LSTM or CNN combined with expert knowledge and the attentional vector.

##### 4.2.5.3. Proposed Models

**Figure 7:**
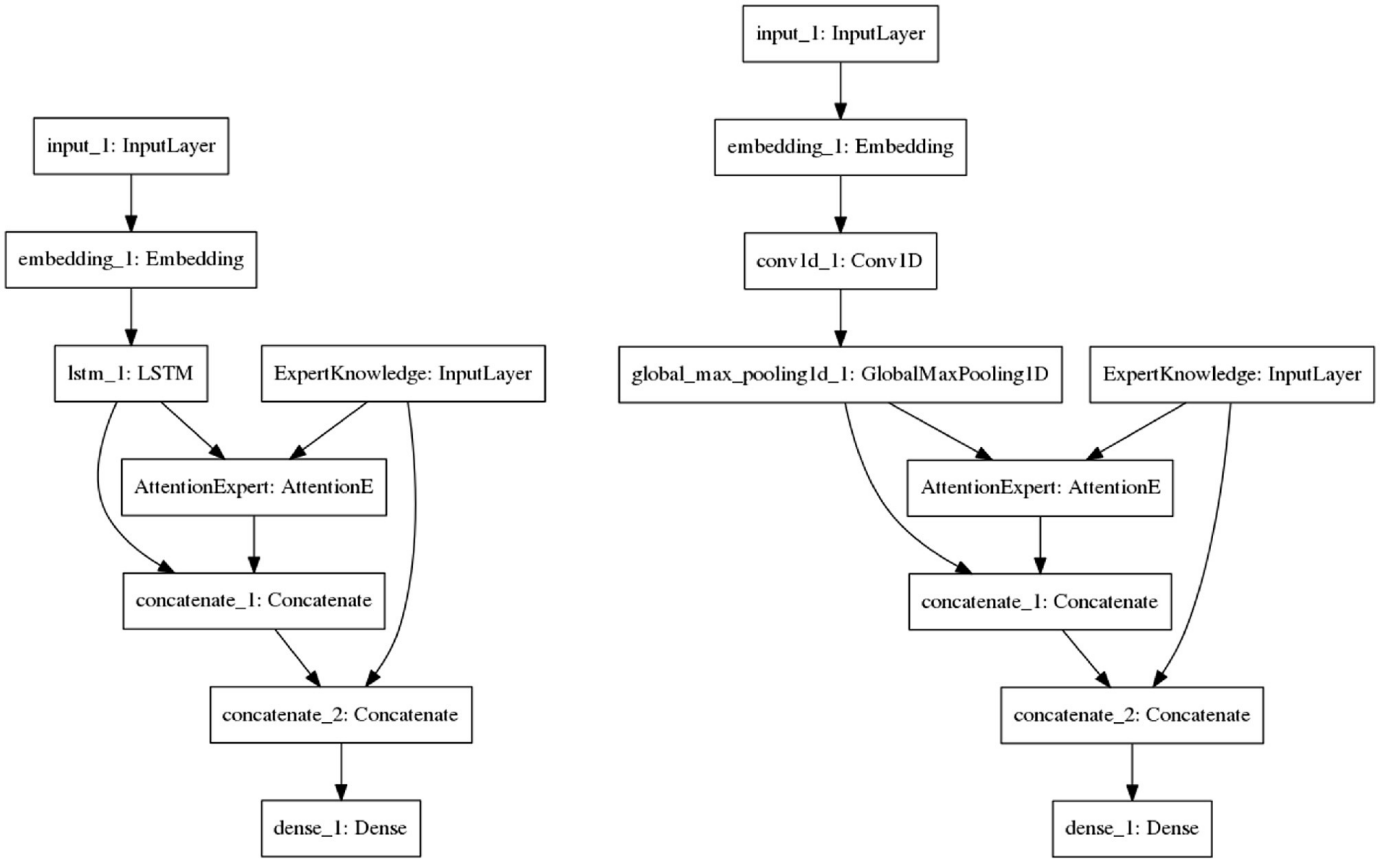
The proposed hybrid architecture using LSTM **(left)** or CNN **(right)** for the prediction of socio-moral reasoning level.

Figure 7 shows the proposed architectures. The models take as input the verbatims that have been pre-processed (tokenization, text to sequence, etc.) and vectored. The vectors are then passed to the embedding layer. In Figure 7 (left), the embedding vectors are passed to the LSTM layer (note that we have only considered the output of the last cell). The expert knowledge and the output of the LSTM are then passed to the expert knowledge-based attention. The attentional vector is merged with the expert knowledge and the output of the LSTM. The concatenation is passed to the last layer for the prediction. This process is the same for the CNN (see Figure 7, right), except that the knowledge-based attention layer takes as input the expert knowledge and the result of the pooling operation applied to the output of the CNN. To evaluate the added value of this knowledge-based attention, we have considered two similar models (cnn-expert, lstm-expert) to those shown in figures. However, those models do not have the knowledge-based attention layer. They are used as a comparison with the proposed solutions.

##### 4.2.5.4. Results

All the models have been trained on 80% (with 20% as validation data) of the data and tested on the remaining 20%. A standard measure for classification/prediction performance is the accuracy. However, for datasets with unbalanced distribution like ours, this measure can be illusory and not very informative on the errors being committed by the classifier. Instead of considering only the accuracy, we have considered the recall, the precision and the F1 score (or F-measure which takes in consideration both the precision and the recall) for all classes and for each of the classes separately. Thus, we will be able to evaluate the performance of the models on the prediction of classes with small samples.

**Figure 8:**
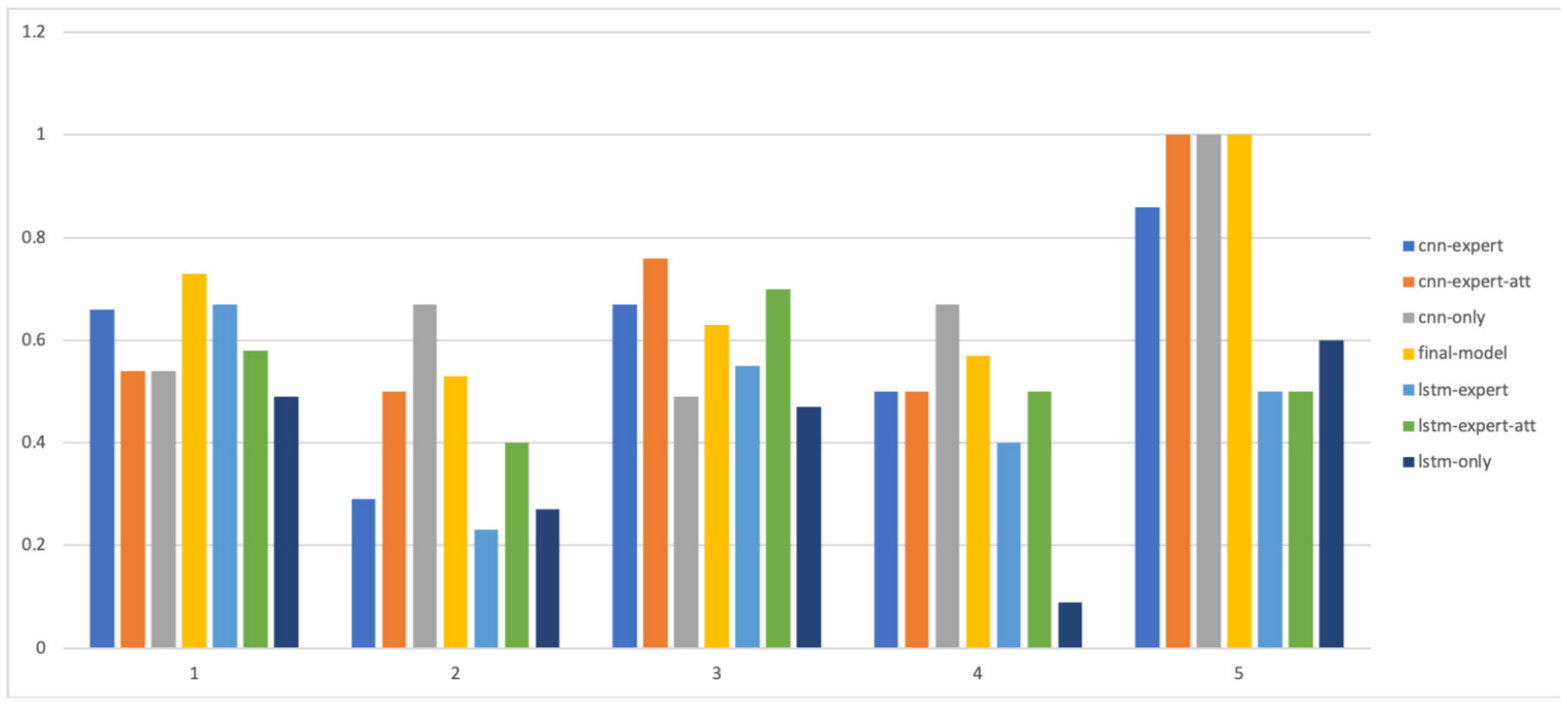
Precision of all the models for each level.

**Figure 9:**
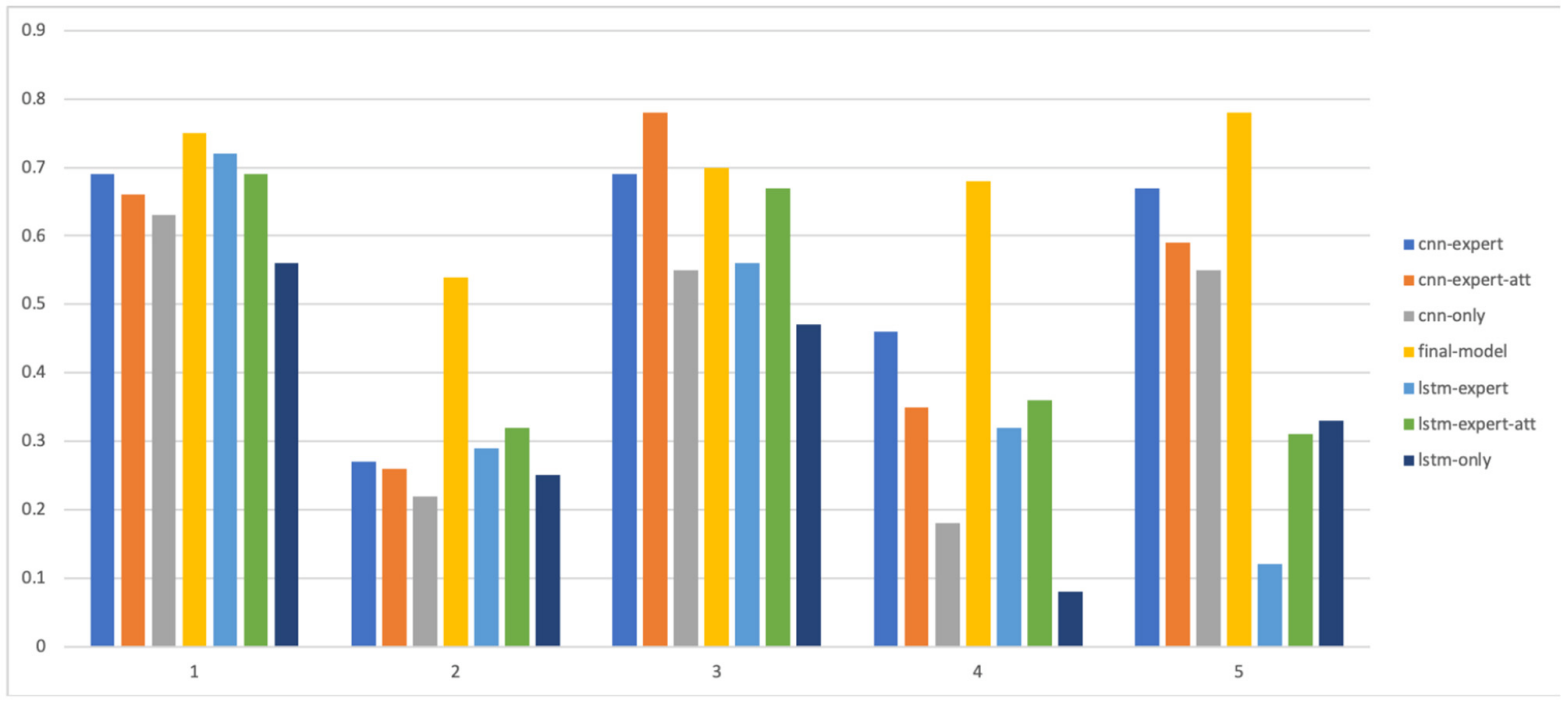
F1-score of all the models for each level.

The results are shown in Table 5 and in Figures 8, 9. As we can see, models that take into account expert knowledge perform well for the prediction of classes with few samples compared to others with no expert knowledge (f1score for classes 2, 4, and 5). This is due to the fact that experts do not need data to make decisions because their decisions are based on their own knowledge. This suggests that, incorporating expert knowledge to neural models improves the classification even when the dataset is unbalanced. Also, even if there is not a huge amount of training data, the models are still able to generalize well on unseen data. Overall, the CNN based models perform well than the LSTM based models because it has been shown that the latter is a solution that has a better generalization power when there is a lot of available data.

**Table 5:** Overall precision, recall, f1score, and accuracy of all the models.

**Models**	**Precision**	**Recall**	**f1-score**	**Accuracy**
Expert-only	0.47	0.40	0.38	0.40
cnn-only	0.58	0.53	0.49	0.53
lstm-only	0.42	0.43	0.42	0.43
cnn-expert	0.62	0.62	0.62	0.62
lstm-expert	0.54	0.53	0.51	0.53
cnn-expert-att	0.67	0.65	0.63	0.65
lstm-expert-att	0.59	0.60	0.58	0.60
Final model	0.72	0.75	0.73	0.75

#### 4.2.6. Discussions

The computed a priori expert knowledge, alone, gave 40% of accuracy on all the dataset, which is not good. However, when directly merged with the features learned by the DNNs, the results are better than without that concatenation. Merging the expert knowledge with the features learned by the models is nonetheless not sufficient as it is comparable to adding a bias. Expert knowledge should be considered as an input that can make change to features learned depending on whether these features are important for the final prediction. As seen in the results, the knowledge-based attention layer helps improve the prediction, which suggests that using the attentional mechanism is suitable for the targeted task. Here are two advantages of incorporating expert knowledge to DNN: (1) Models are able to generalize well even with a limited amount of data; (2) Models are able to make accurate prediction on unbalanced dataset.

##### 4.2.6.1. Real Life Socio-Moral Reasoning Classification

Different experts tend to associate to the same verbatim: different—but close—classes. Taking into consideration that even experts can make errors, we retrained our model by considering a margin error of 1 for the 5-class problem. For example, if the model predicts that the class of the verbatim v is 4 and that the real class is 5, then it is considered as a correct classification. The results taking into account errors margins showed a noticeable improvement. We gained more than 5% on the overall f1score.

##### 4.2.6.2. Personalization in MorALERT

The final model (using CNN as it gave best results) was integrated in the game that is currently in experimentation. The prediction was done in real time. We recorded audio justification of the learner before transforming it to text using Google speech to text API.^8^ The text was then fed into the model, outputting the predicted socio-moral reasoning skill level. The predicted socio-moral reasoning skill level was thereafter used to update the current player model and to adapt the serious game accordingly.

To introduce a form of feedback and scoring inside the game, we added a simulated social feedback showing the number of “likes” and “friends” depending on the player responses. When players' socio-moral reasoning maturity level increased, players gained “likes”, and when they made positive evaluations of the opinions of NPC with a higher level of maturity than their own, they gained “friends”. Their evaluation of the opinions of NPC with lower levels of SMR maturity had no effect on the number of likes or number of friends. For the 10 players we have already experimented, the model was able to correctly predict the socio-moral reasoning skill levels based on the comparison with human experts' prediction. Furthermore, the assessment of the game suggests that the players appreciated it in terms of immersion, playability and impression of having learned something. Results also show that the game encourages the development of higher levels of SMR skill from pre-test compared to the game, but also from pre-test compared to post test. In fact, results during the post-test appear to be lower than during the game, which might be related to the higher level of perceived immersion and also because of the social simulation associated with the NPC and their opinions and also the social feedback interface.

## 5. Conclusion

In this paper, we proposed a simple, effective, and intuitive technique to improve deep learning architecture on user modeling for personalization purposes. It consists of incorporating expert knowledge (when available) using the attention mechanism, to neural network architectures. The solution was tested in two learning environments: Logic-Muse and MorALERT.

In Logic-Muse, the experiments consisted in tracing the logical skills of students using the hybrid model. At the same time, we introduced a new way of using the attention mechanism, to allow neural networks to take into account expert knowledge (when available) in their training and decision process. We tested the solution on a dataset that is unbalanced. The results showed that the DKT is unable to accurately track skills with little data, compare to the DKT+BN.

During the experiments, we noticed that, for skills that are very easy to master, all the 2 models were unable to track incorrect answers, due to the fact that the ratio *r*=incorrect answers/total of questions answered was very low. Even with a penalty, we were not able to significantly improve the DKT model. However, in this paper, we have set the regularization parameters λ_1_ and λ_2_ with a fixed value but for future work, we will do a grid search to find the best values. We will do further experiments on the integration of expert knowledge into neural network architectures. We also plan to test our techniques on a larger and or public dataset.

The proposed model has also been tested in MorALERT, a serious game adaptive learning environment for assessing and optimizing SMR in adolescence. In MorALERT, our model accurately predicts the socio-moral reasoning skill level based on textual verbatim and a priori expert knowledge. This makes it possible to propose an adapted (personalized) order for the dilemma sequencing in the game. The findings from a recent experiment of MorALERT (Zarglayoun et al., 2022) show promise in terms of its potential for assessing and improving socio-moral reasoning maturity.

Thanks to its hybrid nature, the proposed model is also intended to help experts in the annotation of verbatims. Results are very promising for the field. Contrary to the state-of-the-art techniques in text classification, the solution we proposed achieves the best results in our context. This is mainly due to the deep structures that can learn useful features from data and also the a priori knowledge that can leverage unbalanced data.

The idea of using attention to incorporate expert knowledge to NN can be used in other domains such as text classification or in medicine where there is a lot of expert knowledge available. For example, we could think of a classifier using a neural network combined with a rule-based system, as the expert knowledge.

## Data Availability Statement

The raw data supporting the conclusions of this article will be made available by the authors, without undue reservation.

## Author Contributions

AT and RN contributed to conception and design of the study and wrote the sections of the manuscript. AT implemented the systems (Logic-Muse and MorALERT) and the models, organized the datasets used for the experiments, performed the analysis of the results, and wrote the first draft of the manuscript. All authors contributed to manuscript revision, read, and approved the submitted version.

## Funding

This research was supported by grants from Natural Sciences and Engineering Research Council of Canada (NSERC), Discovery Grant Awarded to RN. This research was also supported by grants from Fonds de Recherche du Québec Société et Culture (FRQSC) awarded to RN and his colleagues (Research Team).

## Conflict of Interest

The authors declare that the research was conducted in the absence of any commercial or financial relationships that could be construed as a potential conflict of interest.

## Publisher's Note

All claims expressed in this article are solely those of the authors and do not necessarily represent those of their affiliated organizations, or those of the publisher, the editors and the reviewers. Any product that may be evaluated in this article, or claim that may be made by its manufacturer, is not guaranteed or endorsed by the publisher.

## Footnotes

^1^ shorturl.at/gpxK6

^2^ https://github.com/mmkhajah/dkt

^3^ https://github.com/angetato/Deep-Knowledge-Tracing-On-Skills-With-Limited-Data

^4^ http://fauconnier.github.io/

^5^ http://wacky.sslmit.unibo.it/doku.php?id=corpora

^6^ https://tedboy.github.io/nlps/generated/generated/gensim.similarities.WmdSimilarity.html

^7^ https://keras.io/

^8^ https://cloud.google.com/speech-to-text/
